# Elevated homocysteine is associated with increased rates of epigenetic aging in a population with mild cognitive impairment

**DOI:** 10.1111/acel.14255

**Published:** 2024-06-27

**Authors:** Holly E. Holmes, Rafael E. Valentin, Fredrik Jernerén, Celeste A. de Jager Loots, Helga Refsum, A. David Smith, Leonard Guarente, Ryan W. Dellinger, Dayle Sampson, for the Alzheimer's Disease Neuroimaging Initiative

**Affiliations:** ^1^ Elysium Health New York New York USA; ^2^ From the Oxford Project to Investigate Memory and Ageing (OPTIMA), Department of Pharmacology University of Oxford Oxford UK; ^3^ Department of Pharmaceutical Biosciences Uppsala University Uppsala Sweden; ^4^ Ageing Epidemiology Research Unit, School of Public Health Imperial College London London UK; ^5^ Department of Nutrition, Institute of Basic Medical Sciences University of Oslo Oslo Norway; ^6^ Department of Biology MIT Cambridge Massachusetts USA

**Keywords:** B‐vitamins, DNA methylation, epigenetic age, homocysteine

## Abstract

Elevated plasma total homocysteine (tHcy) is associated with the development of Alzheimer's disease and other forms of dementia. In this study, we report the relationship between tHcy and epigenetic age in older adults with mild cognitive impairment from the VITACOG study. Epigenetic age and rate of aging (ROA) were assessed using various epigenetic clocks, including those developed by Horvath and Hannum, DNAmPhenoAge, and with a focus on Index, a new principal component‐based epigenetic clock that, like DNAmPhenoAge, is trained to predict an individual's “PhenoAge.” We identified significant associations between tHcy levels and ROA, suggesting that hyperhomocysteinemic individuals were aging at a faster rate. Moreover, Index revealed a normalization of accelerated epigenetic aging in these individuals following treatment with tHcy‐lowering B‐vitamins. Our results indicate that elevated tHcy is a risk factor for accelerated epigenetic aging, and this can be ameliorated with B‐vitamins. These findings have broad relevance for the sizable proportion of the worldwide population with elevated tHcy.

## Graphical abstract

This study uses DNA methylation to evaluate the impact of homocysteine (tHcy) levels on epigenetic age and rate of aging (ROA) in older adults with mild cognitive impairment. Elevated tHcy was associated with faster epigenetic aging, and tHcy‐lowering with B‐vitamins normalized accelerated ROAs in individuals with elevated tHcy.

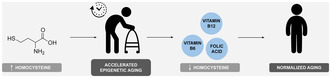

**Abbreviations**
AD: Alzheimer's disease
ADNI: Alzheimer's Disease Neuroimaging Initiative
APOE: gene for apolipoprotein E
BMI: body mass index
CAMDEX: Cambridge Examination for Mental Disorders of the Elderly
DNAm: DNA methylation
HVLT‐R DR: Hopkins Verbal Learning Test—revised with delayed recall
HVLT‐R TR: Hopkins Verbal Learning Test—revised with total recall
MCI: mild cognitive impairment
MMSE: Mini Mental State Examination
MRI: magnetic resonance imaging
PC: principal component
PET: positron emission tomography
POA: pace of aging
ROA: rate of aging
SDMT: Symbol Digit Modalities Test
SRM: Spatial Recognition Memory
tHcy: total homocysteine
TICS‐M: Telephone Interview for Cognitive Status—modified

## 1 INTRODUCTION

Elevated plasma total homocysteine (tHcy) is a modifiable risk factor for cognitive decline, accelerated brain atrophy, and Alzheimer's disease (AD) (Clarke et al., 1998; den Heijer et al., 2002; Elias et al., 2005; McCaddon et al., 1998; Smith, 2008). tHcy levels are regulated by the availability of certain B‐vitamins (vitamin B6, folate, and vitamin B12) which are cofactors or substrates for enzymes involved in tHcy metabolism (Smith et al., 2010). Dietary supplementation with these B‐vitamins has been shown to effectively lower tHcy levels by ~30% in countries without folic acid fortification (Homocysteine Lowering Trialists' Collaboration, 2005).

VITACOG is a randomized, double‐blind, placebo‐controlled study demonstrating that daily administration of B‐vitamins (20 mg vitamin B6, 800 mcg folic acid, and 500 mcg vitamin B12) significantly lowered tHcy and slowed the rate of brain atrophy in subjects with mild cognitive impairment (MCI) (Smith et al., 2010). The brain shrinkage rate over 2 years in the B‐vitamin group was 30% less than in the placebo group (0.76% and 1.08% annual reduction in whole brain volume, respectively). Notably, the effect of the treatment was greatest in subjects in the upper quartile of tHcy at baseline, where whole brain atrophy was reduced by 53% relative to placebo. Thus, tHcy‐lowering with B‐vitamins represents a simple and cost‐effective approach to potentially slow cognitive decline and brain atrophy associated with MCI and perhaps aging and neurodegenerative diseases (Smith & Refsum, 2016).

DNA methylation (DNAm) is a key epigenetic mechanism involving the addition of a methyl group to the fifth carbon of a cytosine residue, typically occurring at cytosine–guanine dinucleotides (termed CpGs sites) in mammals (Jones et al., 2015). Methylation is facilitated by DNA methyltransferases, while the active removal of methyl groups is initiated by the 10–11 translocation family of enzymes; these modifications alter downstream gene expression without changing the underlying DNA sequence, effectively silencing or activating genes depending on the context (Zhang et al., 2023). Operating at the intersection between the genome and environmental factors, DNAm dynamically responds to both intrinsic and extrinsic exposures, such as diet, exercise, UV radiation, and pollutants. Changes in methylation patterns are closely associated with aging and a variety of age‐related diseases, including AD, cardiovascular disease, and cancer.

In recent years, DNAm‐based age predictors, or “clocks,” have emerged as promising biomarkers of aging (Horvath & Raj, 2018; Jylhävä et al., 2017). The first‐generation clocks were trained to predict chronological age from the methylation status of tens to hundreds of CpG sites, e.g., Horvath's 2013 and 2018 clocks (Horvath, 2013; Horvath et al., 2018) and Hannum's clock (Hannum et al., 2013). The remarkable ability of first‐generation clocks to predict all‐cause mortality independent of classic risk factors (e.g., age, body mass index [BMI], and smoking) has led to their rise as unbiased biomarkers of aging (Jylhävä et al., 2017). In recent years, a second generation of clocks have emerged that have been trained as predictors of aging outcomes to yield an “epigenetic age” that is a robust predictor of diverse morbidity and mortality outcomes, including all‐cause mortality; these clocks include DNAmPhenoAge (Levine et al., 2018). Most recently, a third‐generation clock known as DunedinPACE has been developed. DunedinPACE differs from first and second‐generation clocks as it directly outputs a “pace of aging” (POA) based on longitudinal data from a homogeneous cohort (Belsky et al., 2022).

DNAm‐based clocks are beginning to be employed to gauge the effects of lifestyle interventions and experimental therapeutics on epigenetic aging. One example reported the effect of a thymus regeneration protocol, and identified a 1.5‐year reduction in mean epigenetic age, calculated as the average across four different epigenetic clocks (Fahy et al., 2019). Subsequent studies explored the effect of vitamin D supplementation, the Mediterranean diet, and lifestyle modifications (including diet, sleep, exercise, and relaxation protocols) on epigenetic age (Chen et al., 2019; Fitzgerald et al., 2021; Gensous et al., 2020). Most recently, post hoc analysis of the CALERIE (Comprehensive Assessment of Long‐term Effects of Reducing Intake of Energy) trial found that 25% caloric restriction for 2 years slowed the POA, assessed using DunedinPACE (Waziry et al., 2023).

The goal of this study was to determine the effects of homocysteine levels and B‐vitamin supplementation on epigenetic age, using seven published DNAm‐based clocks, with a focus on a new clock, Index. Like DNAmPhenoAge, Index is trained to predict an individual's “PhenoAge” which uses clinical biomarkers to predict age‐related mortality and morbidity risk, but incorporates methods that dramatically improve reproducibility without compromising all‐cause mortality predictions (Higgins‐Chen et al., 2022). Our results show that elevated tHcy can accelerate the rate of epigenetic aging, and, more generally, that Index might be a useful tool for capturing this risk factor associated with declining cognitive function during aging.

## 2 RESULTS

### 2.1 Baseline characteristics of VITACOG participants

We analyzed the DNA of 107 subjects in the B‐vitamin group and 110 subjects in the placebo group from the VITACOG study (Smith et al., 2010). Table 1 presents selected characteristics of these subjects and average epigenetic ages at baseline for the following clocks: Index, DNAmPhenoAge, DunedinPACE, Hannum, Horvath's 2013 and 2018 clocks, Weidner, and Zhang. There were no significant differences in epigenetic ages between the two clinical groups. Interestingly, the mean epigenetic ages for subjects in both groups were lower than their mean chronological age for all the clocks, with the exception of Weidner. We also observed that the POA computed from DunedinPACE was >1, suggesting—on average—faster epigenetic aging.

**TABLE 1:** Baseline characteristics of VITACOG participants.

	Placebo (*n* = 110)		B‐vitamin (*n* = 107)		*p*‐Value
	Mean	SD	Mean	SD	
Age	76.7	4.7	76.9	5.1	0.796
Women, *n* (%)	71	64.5	68	63.6	—
BMI, kg/m^2^	26.2	4	25.7	3.5	0.353
Systolic blood pressure, mmHg	145.7	19.5	147.3	23.5	0.587
Diastolic blood pressure, mmHg	79.8	11.2	79.5	11	0.840
Ever‐smoker, *n* (%)	55	50	45	31.8	—
APOE e4 positive, *n* (%)	35	31.8	33	30.8	—
Years of education	14.8	3.5	14.3	3.5	0.248
Blood measurements					
tHcy, μmol/L	12.2	4.2	11.8	3.5	0.500
Cognitive assessments					
MMSE	28.2	1.4	28.2	1.8	0.915
HVLT‐R DR	7.4	3.2	7.7	3	0.496
Category fluency	19.9	5.2	20.5	4.9	0.331
DNAm‐based clocks					
Index	64.4	7.6	63.9	6.9	0.582
DNAmPhenoAge	61.6	7.6	61.5	8	0.941
DunedinPACE	1.08	0.08	1.09	0.08	0.224
Hannum	60.7	6.4	60.9	6.1	0.830
Horvath 2013	73	6.8	73.4	6.8	0.665
Horvath 2018	71.6	6	71.2	5	0.598
Weidner	92.8	8.6	92.3	9.4	0.659
Zhang	73.7	5.7	73.7	5.2	0.979

*Note*: Epigenetic ages are presented for all the DNAm‐based clocks, with the exception of DunedinPACE which outputs a rate directly. A *t* test was used to determine significant differences between groups.

Abbreviations: APOE, gene for apolipoprotein E; BMI, body mass index; HVLT‐R DR, Hopkins Verbal Learning Test‐revised with delayed recall; MMSE, Mini‐Mental State Examination; tHcy, plasma total homocysteine.

### 2.2 Relationship between homocysteine and ROA at baseline

We sought to identify whether elevated tHcy levels were associated with faster rates of aging (ROAs). The ROA measurement utilized in this analysis involved dividing epigenetic age by chronological age, except in the case of the DunedinPACE clock which outputs POA as a direct estimate of the ROA.

**FIGURE 1:**
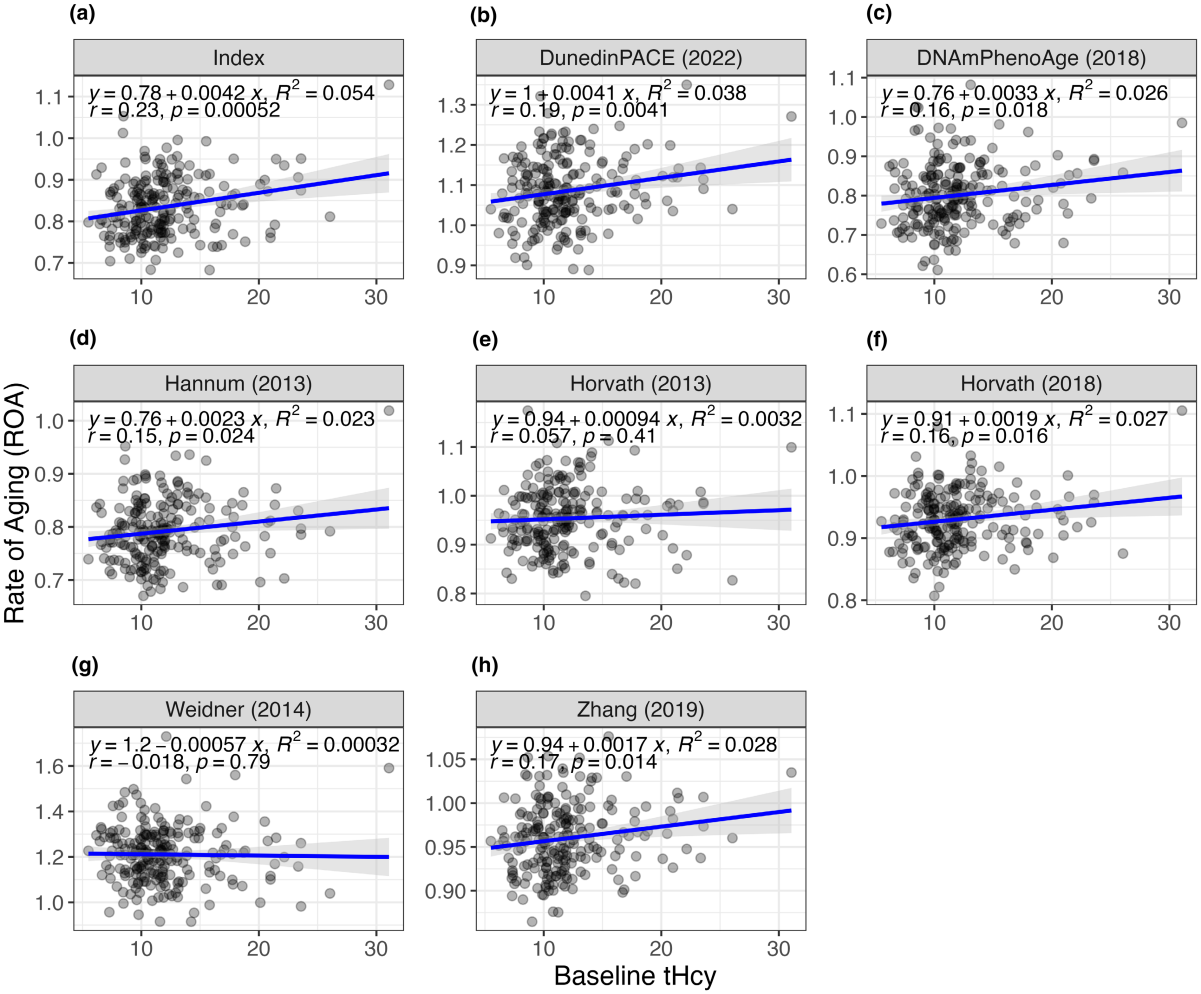
Linear regression and Pearson's correlation of rate of aging recorded at baseline and participant's baseline homocysteine (tHcy) levels. Clocks assessed were (a) Index, (b) DunedinPACE, (c) DNAmPhenoAge, (d) Hannum, (e) Horvath's 2013 clock, (f) Horvath's 2018 clock, (g) Weidner, and (h) Zhang.

Figure 1 shows the results from Pearson's correlations and linear regression analyses between baseline tHcy levels and baseline ROA for all clocks. The majority of clocks showed a positive directional relationship between ROA and tHcy levels at baseline, with faster ROAs observed in subjects with higher tHcy levels (Figure 1). This correlation reached significance for Index (*r* = 0.23, *p* ≤ 0.001), DunedinPACE (*r* = 0.19, *p* ≤ 0.01), DNAmPhenoAge (*r* = 0.16, *p* ≤ 0.05), Hannum (*r* = 0.15, *p* ≤ 0.05), Horvath 2018 (*r* = 0.16, *p* ≤ 0.05), and Zhang (*r* = 0.17, *p* ≤ 0.05). Further analyses using linear regression showed that the variability in ROA could be explained by tHcy levels, accounting between 0.3% and 5.4% of the variance in the ROA delta, as indicated by *R* ^2^ values ranging from 0.00032 to 0.054. Of all the clocks, Index exhibited the highest correlation and lowest *p*‐value. In sum, the baseline data suggests higher tHcy is associated with faster ROAs.

### 2.3 Impact of B‐vitamins on ROA across the entire cohort

Given the significant associations between tHcy levels and ROA at baseline, we subsequently investigated whether lowering tHcy with B‐vitamins would reduce ROA over the 2‐year study period. Treatment with placebo or with the B‐vitamin complex did not significantly change the mean ROA of either clinical group for any of the clocks under investigation (Table 2), except for Weidner which detected a reduction in ROA in both the placebo and B‐vitamin groups. This clock employs only three methylation sites and appears to be subject to greater fluctuations in results (Weidner et al., 2014).

**TABLE 2:** The effect of B‐vitamin treatment on rate of aging (ROA), calculated by dividing each participant's epigenetic age with their chronological age.

	Baseline (BL)				*p*‐Value	Two‐year follow‐up (2 years)				*p*‐Value		
	Placebo (*n* = 110)		B‐vitamin (*n* = 107)		Intragroup: BL	Placebo (*n* = 110)		B‐vitamin (*n* = 107)		Intergroup: 2 years	Intragroup: Placebo	Intragroup: B‐vitamin
	Mean	SD	Mean	SD		Mean	SD	Mean	SD			
Index	0.84	0.07	0.83	0.07	0.408	0.84	0.08	0.83	0.07	0.469	0.915	0.859
DNAmPhenoAge	0.8	0.07	0.8	0.09	0.844	0.81	0.08	0.81	0.08	0.726	0.764	0.396
DunedinPACE	1.08	0.08	1.09	0.08	0.224	1.07	0.09	1.08	0.09	0.445	0.368	0.217
Hannum	0.79	0.06	0.79	0.06	0.884	0.78	0.06	0.78	0.07	0.771	0.246	0.411
Horvath 2013	0.95	0.07	0.96	0.06	0.733	0.94	0.07	0.95	0.07	0.683	0.359	0.335
Horvath 2018	0.93	0.05	0.93	0.04	0.318	0.93	0.05	0.92	0.05	0.285	0.697	0.418
Weidner	1.21	0.12	1.2	0.13	0.582	1.16	0.13	1.16	0.13	0.809	0.001	0.005
Zhang	0.96	0.04	0.96	0.04	0.716	0.96	0.04	0.95	0.04	0.203	0.901	0.217

*Note*: Intergroup analysis was performed using an unpaired two‐tailed *t* test, and intragroup analysis was performed using a paired two‐tailed *t* test.

Abbreviation: ns, nonsignificant.

### 2.4 Impact of B‐vitamins on ROA as a function of baseline tHcy

**FIGURE 2:**
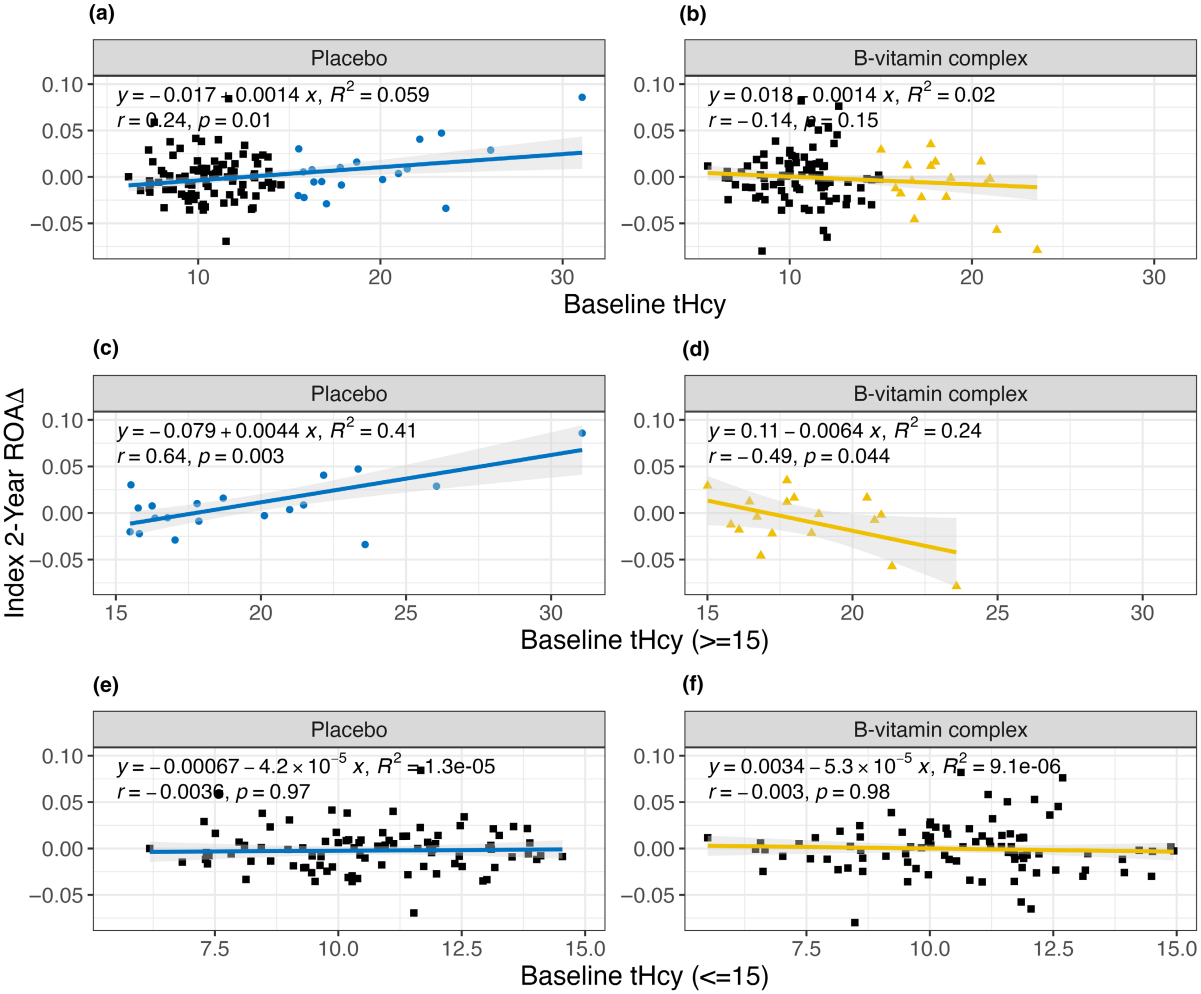
Linear regression of Index's 2‐year change in rate of aging (ROA) and baseline homocysteine (tHcy) levels for (a) the placebo group, and (b) the B‐vitamin group. This analysis was repeated for participants with clinically defined hyperhomocysteinemia (i.e., tHcy ≥15 μmol/L) for (c) the placebo group (blue circles), and (d) the B‐vitamin group (yellow triangles), and for participants with tHcy <15 μmol/L for (e) the placebo group (blue line), and (f) the B‐vitamin group (yellow line).

A previous VITACOG publication reported that treatment response was directly related to baseline tHcy levels: participants in the fourth or highest quartile of tHcy treated with the B‐vitamin complex exhibited a dramatic 53% reduction in rate of brain atrophy, relative to placebo (Smith et al., 2010). These prior findings motivated us to investigate whether the accelerated epigenetic aging in individuals with high tHcy could be modified by the B‐vitamin intervention. We focused on Index, because it best revealed the relationship between tHcy levels and ROA at baseline (Figure 1). In the placebo group, baseline tHcy levels showed a significant positive correlation to the 2‐year change in ROA (*r* = 0.24, *p* ≤ 0.01), consistent with the correlation at baseline (Figure 2a). Approximately 6% of variance in the 2‐year change in ROA was explained by baseline tHcy (*R* ^2^ = 0.059). In the B‐vitamin group, this correlation was not observed (*r* = −0.14, *p* = 0.15, *R* ^2^ = 0.02) (Figure 2b).

We next focused on subjects with hyperhomocysteinemia, which is characterized as tHcy ≥15 μmol/L (Ravaglia et al., 2003, 2005). In this subgroup of the placebo group, the above patterns were amplified and the correlation between baseline tHcy and the 2‐year change in ROA became even more positive with greater significance (*r* = 0.64, *p* ≤ 0.01) (Figure 2c), and over 40% of variability in the 2‐year change in ROA was explained by tHcy (*R* ^2^ = 0.41). However, in this subgroup of the B‐vitamin group, the correlation was actually reversed and became negative (*r* = −0.40, *p* ≤ 0.05) (Figure 2d). These findings suggest that tHcy‐lowering by the B‐vitamins ameliorates accelerated epigenetic aging in subjects with elevated tHcy (Figure 2b), and actually lowers epigenetic age in people with clinically defined hyperhomocysteinemia (baseline tHcy ≥15 μmol/L) (Figure 2d). We also examined subjects with baseline tHcy <15 μmol/L (i.e., in the absence of hyperhomocysteinemia) and no significant correlations were observed, with Pearson's *r* and *R* ^2^ values close to zero (Figure 2e,f). This indicates that the effect of B‐vitamins on ROA may be confined to subjects with elevated tHcy at baseline.

**FIGURE 3:**
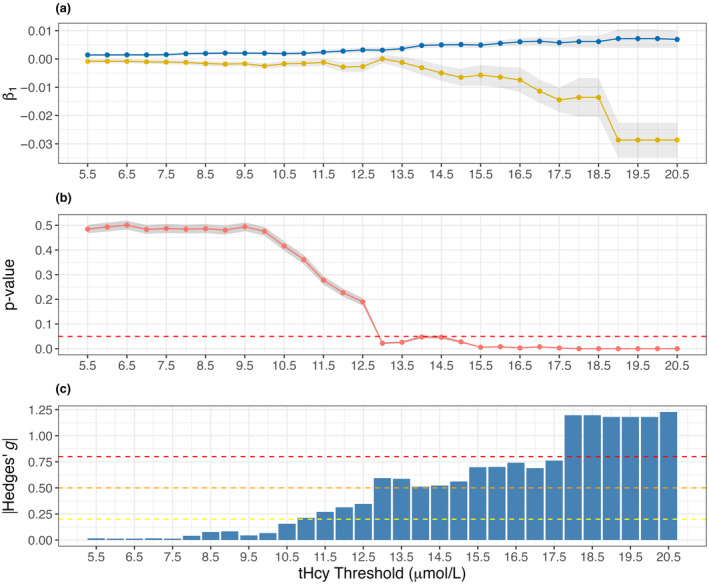
(a) Computed slope changes for placebo (blue) and B‐vitamin (yellow) groups, subset across different baseline homocysteine levels, ranging from 5.5 to 20.5 μmol/L. The *x*‐axis is the minimum homocysteine level in μmol/L, and the *y*‐axis is the recorded slope for each clinical group. The light gray interval represents the standard error of the linear regression model for each point. (b) The mean bootstrapped *p*‐value observed between clinical groups with different minimum thresholds for baseline homocysteine. The red horizontal dashed line indicates *p* = 0.05. The gray shaded areas represent the 95% confidence interval. (c) Hedges' g effect size comparing 2‐year change in rate of aging between placebo and B‐vitamin groups. Yellow, orange, and red dashed lines represent small, medium, and large effect size thresholds, respectively (Sawilowsky, 2009).

To better visualize the trends between tHcy and epigenetic age, the slope for regression lines of baseline tHcy versus the 2‐year change in ROA for each group was calculated across tHcy levels ranging from ≥5.5 μmol/L to ≥21 μmol/L in 0.5 μmol/L increments (Figure 3a). The two groups diverged sharply at 13.5 μmol/L (*p* ≤ 0.05), and reached the greatest separation at 19 μmol/L. However, at higher thresholds of baseline tHcy, the lower number of subjects per group result in a greater standard error, shown by the shaded gray region in Figure 3a, thereby reducing statistical power. Indeed, at the highest threshold of ≥20.5 μmol/L, there are only three and two subjects in the placebo and B‐vitamin groups, respectively. To further characterize the interaction between tHcy and ROA, the between‐group bootstrapped *p*‐values for the B‐vitamin and placebo interventions were calculated for increasing tHcy concentrations (Figure 3b). We found a statistically significant difference between the clinical groups at tHcy ≥13.0 μmol/L, which was maintained at higher tHcy concentrations. This is notable as it suggests that the B‐vitamins may benefit individuals with moderately raised tHcy levels that fall below clinically defined hyperhomocysteinemia. We also observed a sequential effect size increase as the threshold of baseline tHcy increased (Figure 3c).

We conclude from these findings: First, there is an overall positive correlation between baseline tHcy levels and the 2‐year change in ROA in the placebo group, suggesting that elevated tHcy is associated with accelerated epigenetic aging (Figure 2a,c). Second, the B‐vitamin intervention blunts this correlation overall (Figure 2b), and actually reverses it in hyperhomocysteinemic subjects (Figure 2d), most likely due to the tHcy‐lowering actions of the B‐vitamins. Thus, the B‐vitamin complex effectively decelerates epigenetic aging in people with clinically defined hyperhomocysteinemia.

### 2.5 Comparison of index and other clocks for 2‐year change in ROA

**FIGURE 4:**
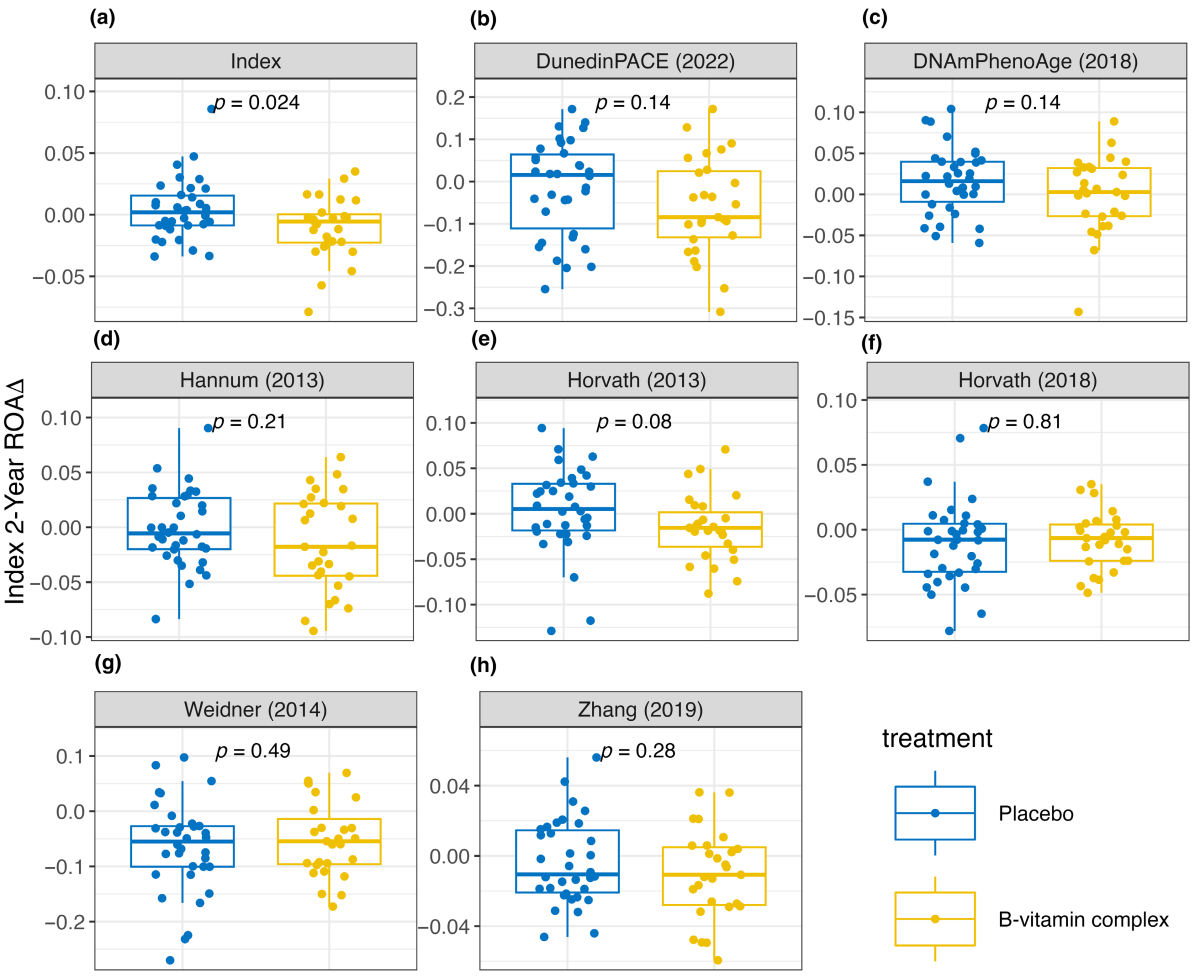
Box and whiskers plots illustrating the 2‐year change in rate of aging (ROA) for participants in the placebo (*n* = 34) and B‐vitamin (*n* = 27) groups with baseline homocysteine levels ≥13 μmol/L. *t*‐Tests were performed to identify significant differences between the placebo and B‐vitamin groups. Clocks assessed were (A) Index, (B) DunedinPACE, (C) DNAmPhenoAge, (D) Hannum, (E) Horvath's 2013 clock, (F) Horvath's 2018 clock, (G) Weidner, and (H) Zhang.

We next sought to investigate whether we could determine a treatment effect in response to the B‐vitamin intervention, relative to placebo, across all clocks. For this analysis, we now focused on subjects with tHcy ≥13 μmol/L, as the bootstrapped *p*‐value analysis identified between‐group differences with Index at this threshold (Figure 3b). The data are now represented by Box and Whisker plots (Figure 4). Most of the eight clocks (Index, DunedinPACE, DNAmPhenoAge, Hannum, Horvath 2013, and Zhang) showed a trend toward a reduction in the 2‐year change in ROA in the B‐vitamin group relative to placebo. However, only Index detected a significant reduction in the 2‐year change in ROA relative to placebo (*p* ≤ 0.05; Figure 4a) in this subgroup with tHcy ≥13 μmol/L.

We then expanded the analysis of between‐group differences across increasing tHcy concentrations—as reported for Index in Figure 3b—to include all the clocks, to determine whether they have significant treatment effects at higher tHcy thresholds. Some of the clocks detected significant between‐group differences at higher tHcy thresholds (Figure S1); this included DNAmPhenoAge and Hannum (both *p* ≤ 0.05 at ≥15.5 μmol/L) and Horvath 2013 (*p* ≤ 0.05 at ≥18 μmol/L). However, their trends toward significance were more stochastic, and some of the clocks transitioned in and out of significance as the tHcy threshold increased. Index was the most sensitive to the B‐vitamin intervention at the lowest tHcy threshold, and demonstrated a steady trend toward statistical significance.

### 2.6 Relationship between ROA and cognitive function

**FIGURE 5:**
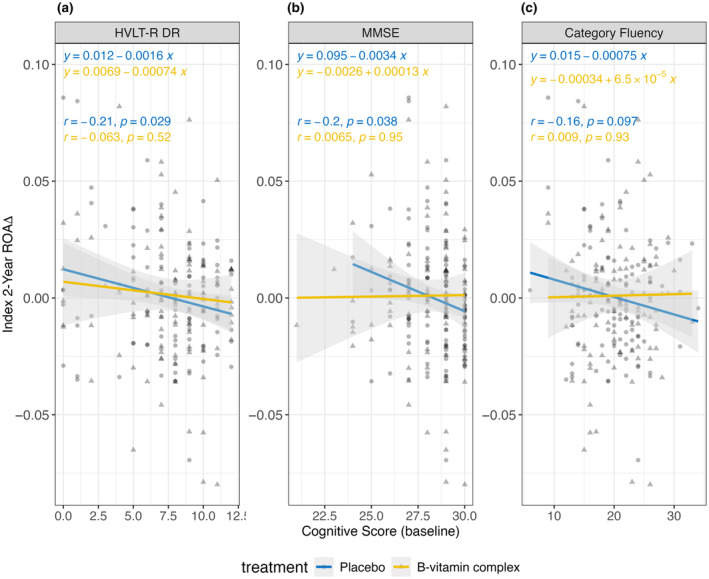
Linear regression and Pearson's correlation of Index 2‐year change in rate of aging (ROA) against baseline cognitive scores for (a) Hopkins Verbal Learning Test Revised‐Delayed Response (HVLT‐R DR), (b) Mini‐Mental State Examination (MMSE), and (c) Category Fluency. The gray shaded area represents the 95% confidence intervals.

Finally, the clinical significance of the 2‐year change in ROA was investigated by assessing its possible association with cognitive function (Figure 5). This analysis focused on assessments that are representative of cognitive domains of importance in MCI: episodic memory (HVLT‐R DR), global cognition (MMSE), and semantic memory (category fluency, CERAD) (de Jager et al., 2012). We first compared baseline cognitive performance with the 2‐year change in ROA, determined using Index (Figure 5). Subjects in the placebo group exhibited significant negative correlations between the 2‐year change in ROA and baseline performance on HVLT‐R DR (*r* = −0.21, *p* ≤ 0.05) and MMSE (*r* = −0.2, *p* ≤ 0.05), with category fluency reaching borderline significance (*p* = 0.097). Strikingly, these correlations were abolished or blunted in the B‐vitamin group (*p* = 0.52, *p* = 0.95, and *p* = 0.93, for HVLT‐R DR, MMSE, and category fluency, respectively). Normalizing to baseline tHcy abolished the effects for category fluency and HVLT‐R DR (Figure S2D–F), suggesting that the relationship between these measures and the 2‐year change in ROA may be driven by baseline tHcy values which are a known risk factor for cognitive decline. MMSE retained statistical significance (*p* ≤ 0.05), suggesting that these observations may be at least partly independent of baseline tHcy. Two other cognitive assessments (of seven tested) also displayed significant correlations with the 2‐year change in ROA in the placebo group, but not in the B‐vitamin group: Hopkins Verbal Learning Test—revised with total recall (placebo group: *p* ≤ 0.001, B‐vitamin group: *p* = 0.42) and graded naming (placebo group: *p* ≤ 0.01, B‐vitamin group: *p* = 0.15) (Figure S3).

Our observations suggest that poor cognitive performance at baseline is a risk factor for accelerated epigenetic aging, which is normalized by the B‐vitamin complex. Collectively, these findings indicate that the relationship between baseline cognitive performance and 2‐year change in ROA is analogous to baseline tHcy and the 2‐year change in ROA (Figure 2).

## 3 DISCUSSION

In this analysis of the 2‐year, placebo‐controlled VITACOG study (Smith et al., 2010), we evaluated the impact of tHcy levels on epigenetic age in people with MCI using DNAm‐based clocks. Of the eight clocks tested, Index demonstrated the greatest sensitivity to the impact of tHcy and B‐vitamins on ROA.

Most of the clocks employed in this study identified a positive association between tHcy and ROA at baseline, with high tHcy associated with accelerated epigenetic aging. Using the Index clock, which showed the strongest association between baseline tHcy and ROA, we also observed that hyperhomocysteinemic subjects in the placebo group exhibited further increases in ROA over the 2‐year trial period. These findings suggest that elevated tHcy results in faster ROA, which is further accelerated over the course of the trial. Strikingly, we found that tHcy‐lowering with B‐vitamins significantly reduced the 2‐year change in ROA in the quartile of subjects with the highest baseline tHcy (≥13 μmol/L). In subjects with tHcy <13 μmol/L, the B‐vitamins had no effect on ROA, which did not change over the course of the trial. These findings are consistent with the published results from VITACOG, which reported the greatest reduction in brain atrophy in subjects with baseline tHcy greater than 13 μmol/L (de Jager et al., 2012; Smith et al., 2010).

Our study also revealed an association between cognitive performance and ROA; specifically, subjects with poorer cognitive performance in global cognition and domains of episodic and semantic memory at baseline exhibited accelerated epigenetic aging over the 2‐year study period, and this was reduced or abolished by the B‐vitamin complex.

With the exception of Index, the seven other clocks did not show significant treatment‐related effects on ROA in subjects with moderately elevated tHcy (i.e., tHcy ≥13 μmol/L). This was, perhaps, unexpected, given that other interventional studies have identified reductions in epigenetic age using clocks reported in this study. For instance, supplementation with 4000 IU and 2000 IU vitamin D for 16 weeks was associated with significant reductions in Horvath's 2013 clock and Hannum's clock, respectively (Chen et al., 2019). This prior trial recruited subjects with suboptimal vitamin D status at baseline, and therefore were primed to respond to the intervention. To wit, this study was substantially shorter in duration than VITACOG, and may have captured acute, transient effects at the shorter 16‐week time point. By contrast, the CALORIE trial did not identify significant changes in DNAmPhenoAge or GrimAge after 12 or 24 months of caloric restriction, although DunedinPACE did show a reduction in ROA (Waziry et al., 2023).

We are not certain why Index outperformed all other clocks in this study, but we have several hypotheses. First, unlike most other clocks, Index is a second‐generation clock and trained to capture all‐cause mortality, which may enhance its sensitivity to interventions that reduce all‐cause mortality risk. Second, Index samples over 100,000 CpG sites and likely captures more of the complexity of the biology of aging. Third, Index reduces this high information complexity into principal components (PCs), which improves the reliability of epigenetic clocks (Higgins‐Chen et al., 2022). Clocks that rely on a smaller subset of CpGs such as Weidner's clock (3 CpGs), Hannum's clock (71 CpG sites), Horvath's 2013 clock (353 CpG sites), and DNAmPhenoAge (513 CpG sites) may be more vulnerable to individual, experimental, and technical variation of individual CpGs. Indeed, by applying the PC methods utilized in the development of Index to these clocks, dramatic improvements in their reliability have been reported (Higgins‐Chen et al., 2022).

Our findings show—for the first time—that VITACOG participants with elevated tHcy display faster ROAs. We believe that high tHcy is causally associated with rapid aging because normalizing tHcy levels with B‐vitamins decelerates biological aging over the 2‐year duration of the trial. We suggest that accelerated aging may help drive the broad spectrum of maladies in people with hyperhomocysteinemia, including cardiovascular and neurodegenerative diseases, as well as cognitive decline (Smith & Refsum, 2021). These findings may have broad implications: an estimated 13.6% of men and 8.7% of women aged 60 years and above in the United States have tHcy level >13 μmol/L (Pfeiffer et al., 2008), putting them at risk for accelerated biological aging and severe health consequences. In countries that have not introduced folic acid fortification, these values are likely to be higher. tHcy can effectively be lowered with B‐vitamins, which represents a safe and cost‐effective approach to potentially reduce all‐cause mortality risk, as well as many other adverse outcomes (Smith & Refsum, 2021) in the broader population.

### 3.1 Study limitations

There is great interest in the development and validation of biomarkers of aging. This study employed different DNAm‐based clocks to determine epigenetic age before and after a B‐vitamin intervention. Yet there are other biomarkers of aging that have shown associations with all‐cause mortality and age‐related outcomes; these include telomere length (Rode et al., 2015), glycans (Krištić et al., 2014), and proteomics and transcriptomics‐derived approaches (Holly et al., 2013; Menni et al., 2015). Although their application to VITACOG is beyond the scope of this article, future studies leveraging these biomarkers would further expand our understanding of the relationship between tHcy and aging.

There are limitations in the methodologies here which warrant discussion. In the interest of presenting the most comprehensive analysis of VITACOG, we elected to include all DNAm‐based clocks that we had access to. This included first, second, and third‐generation clocks. This was important, as the different clocks were developed using different methodologies and offer varying insights into the aging process—from basic chronological age predictors, to more nuanced biomarkers of aging incorporating variables that are predictive of mortality and morbidity. First‐generation clocks, trained exclusively on chronological age, initially gained popularity as unbiased lifespan predictors. The subsequent development of second and third‐generation clocks attempted to improve the predictive capabilities of DNAm‐based clocks, and appear to predict time‐to‐death more accurately than first‐generation clocks (Vasileva et al., 2023). However, the variables used to build these clocks (e.g., C‐reactive protein, which informs Index, DunedinPace, and DNAmPhenoAge) may only be relevant in an aging population such as VITACOG (Vasileva et al., 2023). Thus, the use of several clocks in parallel seems wise.

Several of the clocks were not fully compatible with the custom array employed in this study. This included Horvath's 2013 clock (19 out of 353 CpGs missing from the array), Hannum (6 of 71 CpGs missing), and DunedinPACE (70 out of 173 CpGs missing). Further analysis indicated the signal overlap due to missing CpGs was sufficient for Horvath 2013 and Hannum (*r* = 0.999 and *r* = 0.998, respectively); however, DunedinPACE had a *r* = 0.901 which corresponds to ~18% loss in signal, which may have impacted its performance. This study also included the Weidner clock, which computes biological age from just three CpGs (Weidner et al., 2014). While this makes it an incredibly simple biomarker (and the leanest one employed in this study), its simplicity may have reduced its reliability. Indeed, Weidner was one of two clocks that failed to identify a correlation between tHcy and ROA at baseline. Furthermore, Weidner identified a significant reduction in ROA in both the placebo and B‐vitamin groups over the 2‐year study period. This is unlikely to be biologically meaningful. In contrast, Index uses over 100,000 CpGs to calculate epigenetic age and demonstrated the greatest sensitivity to the tHcy‐lowering effects of B‐vitamins in this study.

Finally, we acknowledge that this is a retrospective analysis. Future, prospective studies designed to investigate the impact of B‐vitamins on epigenetic age in people with elevated tHcy are clearly warranted.

## 4 METHODS

### 4.1 Baseline characteristics and assessing 2‐year change in rate of aging among clinical groups

This retrospective study was conducted as a part of the VITACOG trial (registered at www.controlled‐trials.com as ISRCTN94410159). This study received approval from the Oxfordshire National Health Service research ethics committee A (COREC 04/Q1604/100) and was carried out according to the principles of the Declaration of Helsinki. This study recruited subjects with a diagnosis of amnestic or non‐amnestic MCI according to Petersen's criteria (Petersen et al., 2009). MCI is an early stage of cognitive decline and is a risk factor for developing Alzheimer's disease or other forms of dementia. Subjects were screened for their eligibility using the Telephone Interview for Cognitive Status‐modified (TICS‐M) (Brandt et al., 1993) and a category fluency test (Morris et al., 1989). The MCI diagnosis was confirmed using other cognitive tools such as the Mini‐Mental State Examination (MMSE) (Folstein et al., 1975) and the Cambridge examination for mental disorders of the elderly (CAMDEX) (Roth et al., 1986). Additional inclusion criteria included aged 70 years or older, and a study partner available as an informant. Exclusion criteria included a diagnosis of dementia or being treated with anti‐dementia drugs, or taking folic acid (>300 μg/day), pyridoxine (>3 mg/day), or vitamin B12 (>1.5 μg/day) orally. Subjects taking B‐vitamins below these levels were eligible for this study. A detailed description of the VITACOG protocol, including the complete inclusion and exclusion criteria, has been published previously (Smith et al., 2010). Subjects were randomized to one of two clinical groups: the B‐vitamin group (*n* = 138) or placebo group (*n* = 133). The inclusion of a placebo group allows us to control for global shifts in methylation bias that may have otherwise confounded our results. Five subjects withdrew from the study before the start of the trial, and a total of 266 subjects started treatment (*n* = 133 in the treatment group, and *n* = 133 in the placebo group). The treatment group received a daily B‐vitamin complex consisting of vitamin B6 (20 mg, as pyridoxine HCl), folic acid (800 mcg), and vitamin B12 (500 mcg, as cyanocobalamin). The treatment period was 2 years. There were 43 subjects that withdrew from the study; the reasons for withdrawal included diagnosis of cancer and withdrawal of consent, and have been previously described (Smith et al., 2010). A total of 223 subjects finished the trial: *n* = 110 in the treatment group, and *n* = 113 in the placebo group. In this study, we included participants who had completed the trial and had sufficient DNA for methylation analysis at both time points.

### 4.2 Plasma homocysteine

At baseline and after 2 years, non‐fasting blood samples were collected by venipuncture. Plasma tHcy was measured by fluorescence polarization immunoassay using the Abbott IMx fully automated immunoassay analyzer.

### 4.3 DNA methylation

This study utilized whole blood samples from VITACOG participants which were banked at the University of Oxford. Samples were shipped on dry ice and analyzed by Tempus Labs, Inc. (Peachtree Corners, GA). DNA was extracted using the Biomek i7 Automated Workstation (Beckman Coulter) in combination with Qiagen's MagAttract Blood DNA Isolation Kit (Part #940065) following the manufacturer's recommended protocols. To ensure sufficient DNA for analysis, DNA was quantified using PicoGreen and Quantit BR technologies. Five hundred nanogram of DNA was bisulfite converted overnight using Zymo's EZ DNA Methylation Kit. Two hundred nanogram of converted DNA was used for methylation analysis. DNA methylation profiles were measured using a custom Illumina 250 K array developed by Elysium Health and Illumina. The custom array contains all Infinium I probe targets with additional probes that were chosen based on their technical robustness, and to ensure representative coverage across the genome. The array was processed according to the manufacturer's instructions. Signal intensity was measured with the Illumina iScan system to generate beta values, a measure of the degree of methylation at a locus.

### 4.4 Relationship between baseline homocysteine and baseline ROA

To calculate rate of aging (ROA), epigenetic age for each participant was calculated using Index—a new clock that has been commercialized by Elysium Health. Enquiries about using Index should be directed to research@elysiumhealth.com. The following published DNAm‐based clocks were also employed: Horvath's 2013 and 2018 clocks (Horvath, 2013; Horvath et al., 2018), Hannum (Hannum et al., 2013) (Hannum et al., 2013), Weidner (Weidner et al., 2014), Zhang (Zhang et al., 2019), Levine's DNAmPhenoAge (Levine et al., 2018), and DunedinPACE (Belsky et al., 2022). For clocks that were missing CpG sites on the custom Illumina array (Table S1), we assessed the signal overlap, including only those in the analysis with a minimum Pearson's correlation coefficient of 0.9 (Figure S4). Data used to assess the robustness of the clock when missing CpGs were present were obtained from the Alzheimer's Disease Neuroimaging Initiative (ADNI) database (adni.loni.usc.edu), GSE87571 (Johansson et al., 2013), and GSE73115 (Tan et al., 2016). The ADNI was launched in 2003 as a public–private partnership, led by Principal Investigator Michael W. Weiner, MD. The primary goal of ADNI has been to test whether serial magnetic resonance imaging (MRI), positron emission tomography (PET), other biological markers, and clinical and neuropsychological assessment can be combined to measure the progression of mild cognitive impairment (MCI) and early Alzheimer's disease (AD). For up‐to‐date information, see www.adni‐info.org. To determine ROA, epigenetic age was divided by chronological age for all clocks except the DunedinPACE clock which outputs a rate directly. The baseline tHcy (i.e., tHcy at the first clinical visit, prior to intervention) for each participant was regressed against ROA for each of the eight clocks, and a Pearson's correlation was then calculated. Regressions were plotted in facets by clock (Figure 1) using ggplot2 v3.4.0 (Wickham et al., 2016) in R v4.2.1 (R Core Team, 2023).

### 4.5 Baseline homocysteine levels and its relationship with the 2‐year change in ROA

The 2‐year change in ROA for Index was calculated by taking the delta of ROA at time point 2 and time point 1. Positive 2‐year change indicates accelerated ROA, and negative 2‐year change indicates decelerated ROA. Linear regression and Pearson's correlation of the 2‐year change in Index ROA and baseline tHcy concentrations for each participant was calculated and plotted for the B‐vitamin and placebo groups, again using ggplot2 in R (Figure 2a,b) with Index's 2‐year change in ROA as the dependent variable. Patients with baseline tHcy concentrations indicative of hyperhomocysteinemia (≥15 μmol/L) were subset and reanalyzed to assess their relationships between the B‐vitamin and placebo groups (Figure 2c,d). This analysis was repeated for participants with tHcy <15 μmol/L (Figure 2e,f).

To assess the impact of tHcy across a range of concentrations, the linear regression was run at minimum inclusion levels ranging from 5.5 to 20.5 μmol/L at 0.5 μmol/L intervals, with the slope and model standard error for each clinical group recorded at each level (Figure 3a). Statistical significance was determined by bootstrap sampling 1000 times at *n* = 50 for each tHcy threshold, and then a two‐tailed *t* test was used to assess for between‐group differences. Means and 95% confidence intervals from the 1000 bootstraps were calculated (Figure 3b). In addition, Hedge's *g* was used to measure the standardized effect size in 2‐year change in ROA between placebo and the B‐vitamin group (Figure 3c). Visualizations of these analyses were then plotted in ggplot2 to visualize their trend; for Hedge's g the absolute value was plotted.

### 4.6 Significance of homocysteine and ROA change relationship between clinical groups

Significance of the observed differences between the two clinical groups across the tested range of tHcy concentrations was assessed for all previously tested biological clocks using a *t* test. *p*‐Values of the differences between both groups for each clock at each concentration were recorded and plotted in facets using ggplot2 in R. A *n* ^3^ polynomial regression was included to visualize the trend of *p*‐values across tHcy concentrations.

### 4.7 Visualizing impact of B‐vitamin complex on ROA in subjects with elevated homocysteine at baseline

Participants with a tHcy concentration ≥13 μmol/L were selected as it was the lowest value where any of the assessed clocks produced a significant result. Box and whisker plots of each clinical group's 2‐year change in ROA for each DNAm‐based clock were generated in facets using ggplot2 in R to visualize the difference. *t* Tests were conducted for each facet to display the *p*‐values of each clock at the set tHcy concentration.

### 4.8 Relationship between ROA and cognitive outcomes

The relationship between the 2‐year change in Index ROA and several baseline cognitive tests were assessed using robust linear regression and Pearson's correlation. Descriptions of the cognitive tests used in VITACOG have previously been published (de Jager et al., 2012; Perła‐Kaján et al., 2021). The following cognitive tests were included in this study: Category Fluency, Hopkins Verbal Learning Test Revised (HVLT‐R) with Delayed Recall (DR) and Total Recall (TR), Mini‐Mental State Examination (MMSE), CANTAB Spatial Recognition Memory (SRM), Graded Naming Test, Trail Making A and B, Symbol Digit Modalities test (SDMT), and Map Search.

Robust regression and Pearson's correlation was carried out in facets for each cognitive test using ggplot2 in R, with Index's 2‐year change in ROA as the dependent variable for both analyses.

Category Fluency, HVLT‐R DR, and MMSE were selected for Figure 5, while the remainder were included in Figure S2.

## AUTHOR CONTRIBUTIONS

H.E.H., R.E.V., A.D.S, L.G., R.W.D., and D.S. contributed to the conception of this study and the study design. R.E.V. completed the data analysis. R.W.D. and D.S. supervised the analysis. All the authors contributed to the data interpretation. F.J., C.A. de J.L., H.R., and A.D.S. contributed meaningful insights into the VITACOG study. H.E.H. drafted this article, and all authors critically revised and approved the final submitted version.

## FUNDING INFORMATION

This new work was funded by Elysium Health. The original VITACOG trial was funded by the organizations listed previously (Smith et al., 2010).

## CONFLICT OF INTEREST STATEMENT

Index is available to purchase via the Elysium Health website. H.E.H., R.E.F., L.G., R.W.D., and D.S. are employees of Elysium Health and have stock options in the company. A.D.S. and H.R. are named as inventors on two patents held by the University of Oxford on the use of B‐vitamins to treat cognitive disorders (US9364497 and US10966947). FJ is named as an inventor on US10966947. Elysium Health holds the exclusive global rights to these patents and has commercialized this technology as a dietary supplement. ADS is also on Elysium Health's Scientific Advisory Board.

## Supporting information


Appendix S1.

## Data Availability

This data analyzes epigenetic data from the VITACOG study that is proprietary to Elysium Health. The following GEO datasets were used to validate the compatibility of clocks missing CpGs with the custom Illumina array: GSE87571 (Johansson et al., 2013) and GSE73115 (Tan et al., 2016). Data from the Alzheimer's Disease Neuroimaging Initiative (ADNI) were also used for these validations.
