# Supplementary material for: Elevated homocysteine is associated with increased rates of epigenetic aging in a population with mild cognitive impairment
Source: Aging Cell. 2024 Jun 27;23(10):e14255. doi: 10.1111/acel.14255 (PMC11464110; doi:10.1111/acel.14255)
Supplement: Supplementary file 1 — Appendix S1. [file ACEL-23-e14255-s001.docx]

**SUPPLEMENTARY FIGURES AND TABLES**

| **Name** | **No. of CpGs in final algorithm** | **Trained to predict** | **Statistical model** | **No. of CpGs missing from the custom array** |
| --- | --- | --- | --- | --- |
| Hannum (2013) | 71 | Chronological age | Penalized regression (Elastic net) | 6 |
| Horvath (2013) | 353 | Chronological age | Penalized regression (Elastic net) | 19 |
| Horvath (2018) | 391 | Chronological age | Penalized regression (Elastic net) | 0 |
| Weidner (2014) | 3 | Chronological age | Multivariate linear regression | 0 |
| Zhang (2019) | 514 (EN clock) | Chronological age | Elastic net regression | 0 |
| DNAmPhenoAge (2018) | 513 | All-cause mortality risk | Cox penalized regression  (Elastic net) | 0 |
| DunedinPACE (2022) | 173 | All-cause mortality risk | Penalized regression (Elastic net) | 70 |

**Supplementary Table 1** Summary of the published clocks used in this paper.


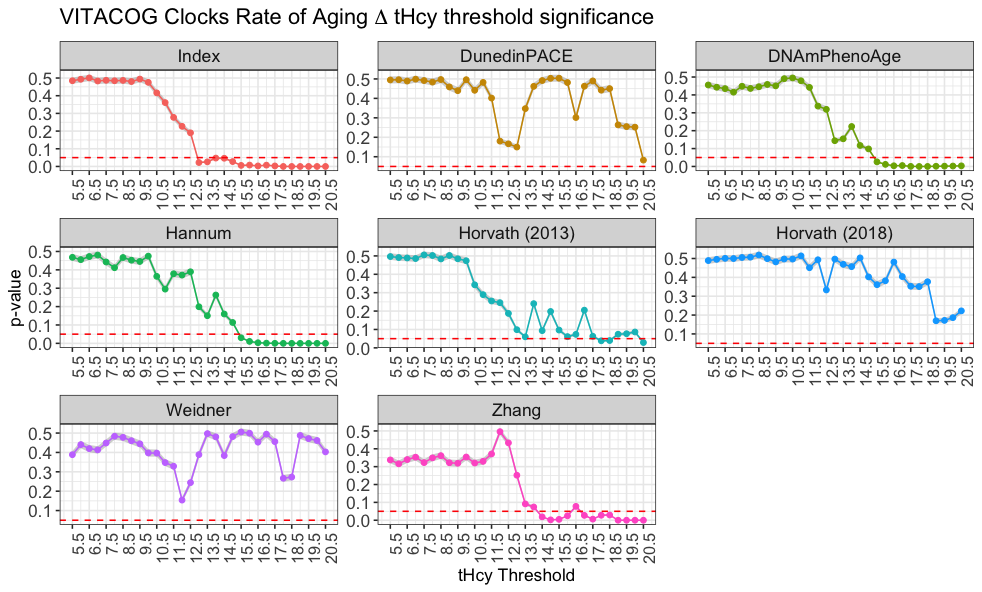


**Supplementary Figure 1** Results (*p*-values) of statistical differences observed between clinical groups with different minimum thresholds for baseline homocysteine (tHcy). The red dashed line indicates *p* = 0.05. DunedinPACE, Horvath (2018) and Weidner fail to detect a significant difference between the placebo and B-vitamin groups at any applied threshold of baseline tHcy. Index, DNAmPhenoAge, Hannum, Horvath (2018) and Zhang detected a pattern of statistical significance (p < 0.05) between groups as the tHcy increased; however, Index reached significance at the lowest tHcy threshold compared to the other clocks.


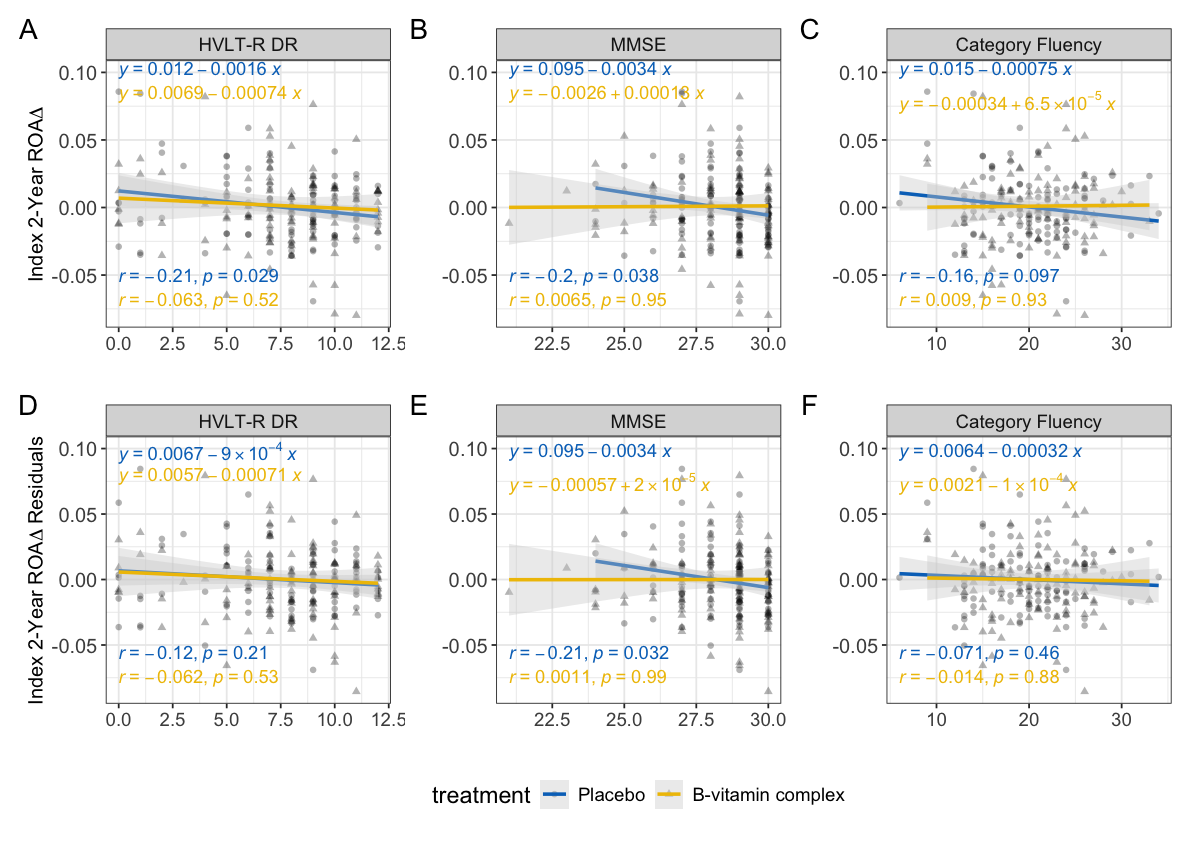


**Supplementary Figure 2** Change in linear regression and Pearson correlation of the two-year change in rate of aging (ROA), determined using Index, against baseline cognitive scores, following normalization to baseline homocysteine (tHcy). (A – C) Relationship between ROA and cognitive performance without tHcy normalization, and (D – E) with tHcy normalization. The gray shaded area represents the 95% confidence intervals.


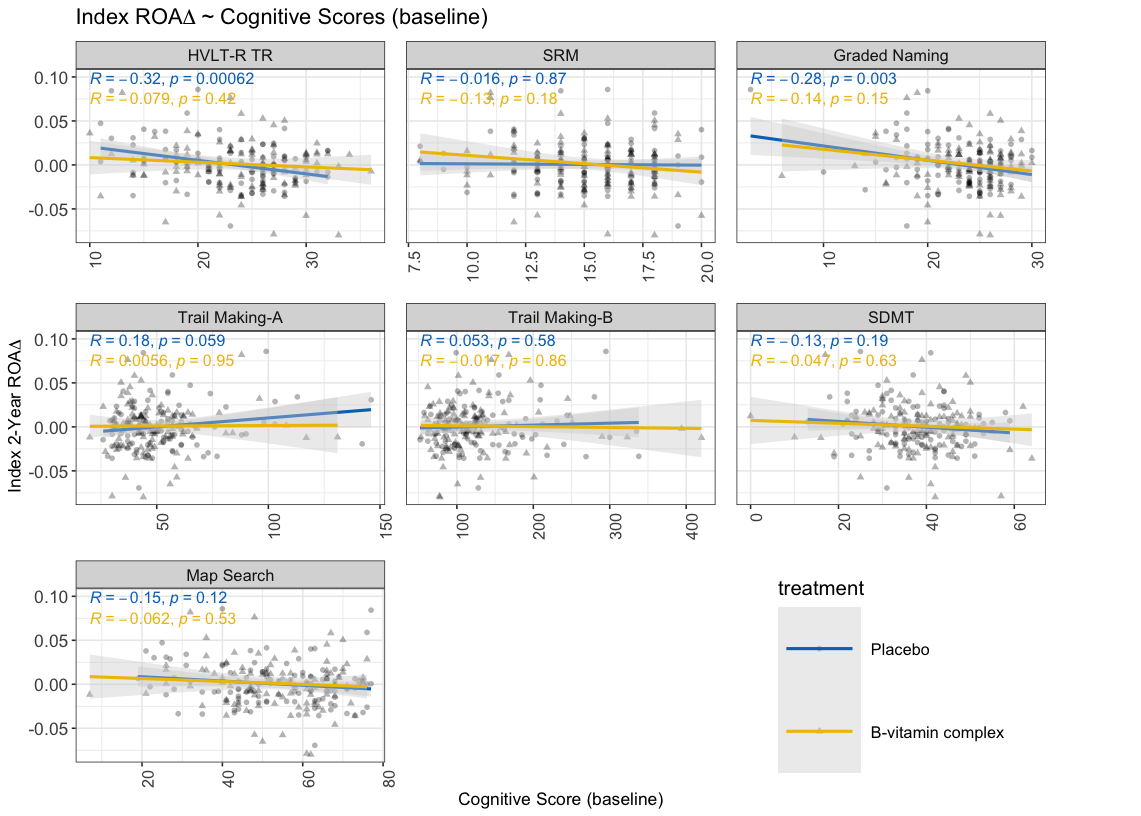


**Supplementary Figure 3** Linear regression and Pearson correlation of the 2-year change in rate of aging for Index against baseline cognitive scores for additional baseline cognitive test scores. The gray shaded area represents the 95% confidence intervals.


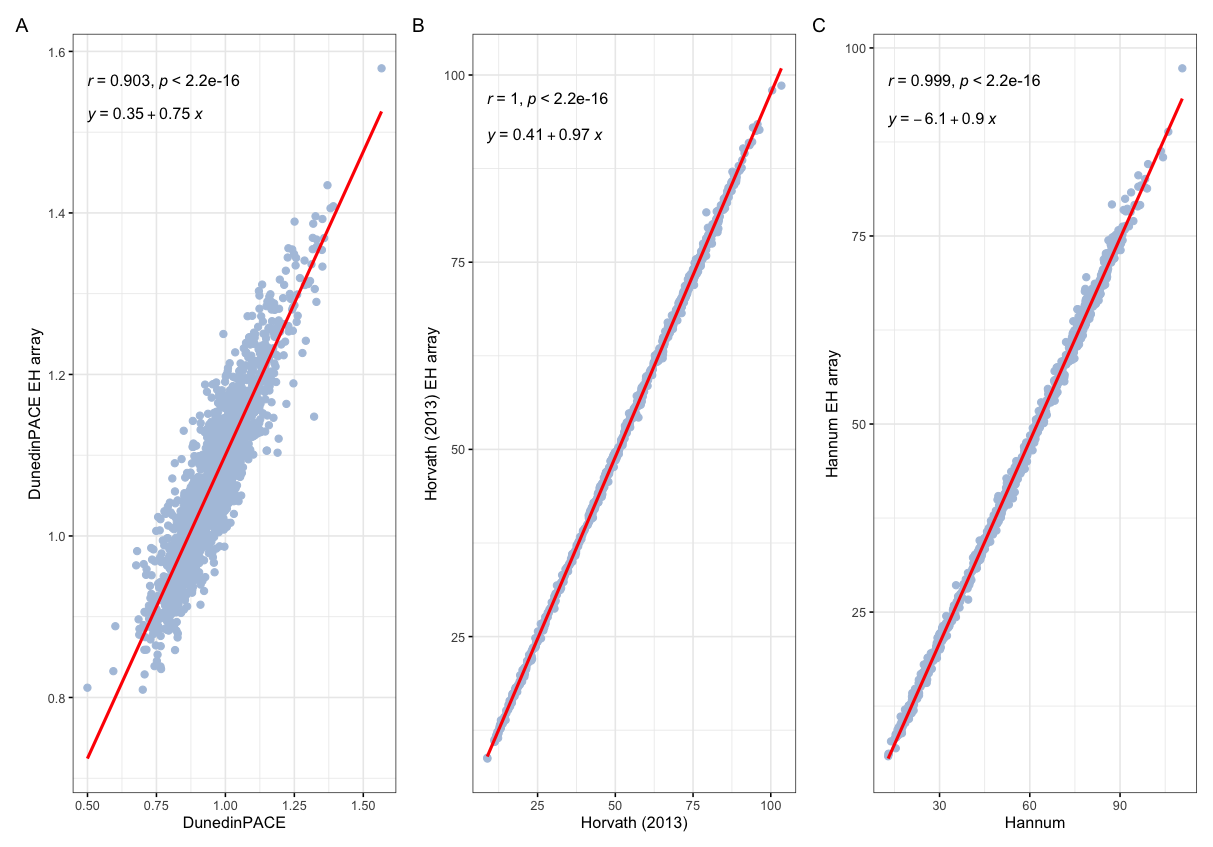


**Supplementary Figure 4** Characterization of signal preservation in DNAm-based epigenetic clocks due to missing CpGs. Three epigenetic clocks had missing CpGs when their algorithms were implemented on a custom methylation array that VITACOG was assessed with: DunedinPACE, Horvath’s 2013 clock, and Hannum’s clock. To determine the overlap in signal a correlation coefficient was calculated in R for each clock. Because the different clocks were developed on different arrays it was important to ensure all CpGs were present, thus the DunedinPACE clock comparison was made using the Illumina EPIC 850K array data from the Alzheimer’s Disease Neuroimaging Initiative (ADNI) while the Horvath and Hannum clocks were compared in the Illumina 450K arrays using GEO datasets GSE87571 (Johansson et al., 2013) and GSE73115 (Tan et al., 2016). The correlation coefficient provides a quantitative measure of the impact of missing CpG sites on each clock's performance. For a clock to be included in subsequent analyses, we required a minimum correlation of 0.9 indicating a very strong positive linear relationship. This threshold ensures that only clocks with less than 18% unexplained variance by the model are included. This decision was grounded in the principle of preserving signal integrity; clocks with a Pearson correlation coefficient less than 0.9 were deemed to retain sufficient reliability for meaningful interpretation. This supplementary figure visually represent the impact of information loss due to missing CpG sites on each clock's performance. We observed a Pearson’s r of 0.903 in the DunedinPACE clock (Figure A). Despite this moderate loss, the clock's overall performance warranted inclusion in our study, as the signal retained was deemed substantial. Horvath’s 2013 clock (Figure B; r = 0.999) and Hannum’s clock (Figure C; ***r*** = 0.998) exhibited very strong signal preservation demonstrating robust performance on the custom array and reaffirming their reliability even in the context of missing CpG sites on the custom array.
